# Assessing the psychometric properties and the perceived usefulness of the BasisRaadsOnderzoek (BARO) as a first-line screening instrument for juvenile offenders

**DOI:** 10.1186/1753-2000-5-24

**Published:** 2011-06-29

**Authors:** Theo AH Doreleijers, Cyril Boonmann, Erik van Loosbroek, Robert RJM Vermeiren

**Affiliations:** 1 Department of Child and Adolescent Psychiatry, VU University Medical Center Amsterdam, The Netherlands; 2 Faculty of Law, Leiden University, The Netherlands; 3 Department of Psychology, University of Maastricht, Maastricht, The Netherlands; 4 Department of Child and Adolescent Psychiatry, Leiden University Medical Center, Leiden, The Netherlands

## Abstract

**Background:**

The aim of this study is to investigate the psychometric properties and the perceived usefulness of the BARO (Dutch: BAsisRaadsOnderzoek; Protection Board Preliminary Examination of Juvenile Suspects). The BARO is a first-line screening instrument for the identification of psychiatric disorders, adverse environmental factors, and levels of (dys)function in adolescent offenders (age 12 to 18), to be used by social workers of the Child Protection Board (CPB) following a police arrest.

**Method:**

CPB workers administered the BARO to 295 juvenile offenders (91% boys, 9% girls). A subgroup of 66 offenders (89% boys, 11% girls) underwent an elaborate diagnostic assessment by forensic psychologists and psychiatrists. Using these assessments the most relevant psychometric properties of the BARO were studied. The perceived usefulness was studied using questionnaires to be filled in by the CPB social workers.

**Results:**

The internal consistency of the instrument was sufficient to good, the concurrent validity of the CPB social workers applying the BARO and the forensic experts carrying out the comprehensive diagnostic assessment was strong, the discriminatory value of the instrument was moderate to strong, and the perceived usefulness of the instrument was evaluated as good to very good by the majority of the CPB workers.

**Discussion:**

The BARO has sufficient to good psychometric properties including moderate to strong discriminatory value and is considered a good screening instrument by the CPB social workers. In conclusion, the BARO seems to be a very promising first-line screening instrument to identify psychiatric and psychosocial problems in young offenders.

## Background

In the Netherlands, approximately 20,000 adolescents (12-18 years) were arrested each year between 1996 and 2000 suspected of committing an offense [1]. These offenses were considered serious enough for formal police registration, legally resulting in an arraignment by the public prosecutor and a referral to the Child Protection Board (CPB). Based on the screening of the CPB, juvenile court can decide on an extensive examination by the Netherlands Institute for Forensic Psychiatry and Psychology (NIFP). Using the assessment of the CPB and the NIFP, juvenile court will determine the sentence. Similar to most other Western European countries, the Netherlands has adopted a criminal law system that treats minors separately from adults. Apart from sanctions, Dutch juvenile criminal law focuses primarily on educational aspects that aim at restoring the juvenile's development. Therefore, the CPB has the legal obligation to make a global assessment of the psychosocial condition of the juvenile in order to advise the judicial authorities concerning further procedures (e.g. an extensive diagnostic assessment) and, if indicated, immediate professional assistance. Because of a lack of standardization among CPB workers in collecting information, an initiative was taken to develop an instrument with the capability of identifying youths at risk for psychiatric disorders and serious psychosocial problems [2].

Earlier research showed that psychosocial adversity is different for offenders and non-offenders, as well as for various groups of offenders [3-5]. Moreover, psychiatric disorders and psychosocial dysfunction are predictive for future delinquency and reoffending [6-9]. To reduce reoffending, it is of great importance to identify those juvenile offenders suffering from psychiatric disorders and psychosocial problems, and treat them in line with their needs. In their responsibility to prevent recidivism and stimulate a positive development of the youth at risk, child welfare services (e.g. CPB) should be able to identify risk and protective factors for psychiatric disorders and psychosocial problems in juvenile offenders. For this purpose a valid and reliable first-line screening instrument useful to CPB workers was needed. The assessment by the CPB worker should provide the judicial authorities necessary information for standardized court decision taking (e.g. immediate punishment, further police investigation, continuation/suspension of the imprisonment). The instrument should be able to recognize individuals in need of immediate help and/or extensive assessment. For such purposes it is important to identify cases accurately (sensitivity: the ability to identify true positives) and to avoid recognizing non-cases as cases (specificity: the ability to identify negative results) [10]. In addition, the procedures must be transparent, they should be carried out within a couple of days, and the investigator-based variability should be minimal. The aim of the BARO project (Dutch: BAsisRaadsOnderzoek; Protection Board Preliminary Examination of Juvenile Suspects) was to develop an instrument that offers more than a numerical risk score. Because the result has to guide the decision-making process of the judicial authorities, it should be a comprehensive - and still easy to compose - evidence based report describing the adolescent's functioning and dysfunction in his/her life.

### Development of the BARO

The BARO was developed using risk factors well known from the literature and by means of secondary analyses of data from a psychiatric prevalence study in adjudicated adolescents [11]. In that study, sociodemographic (age, sex, ethnicity, living situation, parental work/profession level, family size) and offense related data (charges, former convictions, seriousness and damage/injury as a result of the offense) had been collected in a representative group of 108 adolescents (99% boys, 1% girls) 12 to 18 years old. In order to assess psychiatric disorders, screening instruments like the CBCL [12] and the YSR [12], and standardized psychiatric interviews (Child Assessment Schedule (CAS; [13]); the Graham-Rutter Interview; [14]; parts of the translated 'Juvenile Justice Assessment Inventory' (JJAI; [15])) had been administered. To determine intelligence a non-language-sensitive intelligence test, the Raven Progressive Matrices [16], was applied.

First, a semi-structured interview was constructed, which covered nine domains of development, psychiatric disorders and psychosocial (dys)function, based on the topical literature: delinquent behaviour, physical condition, psychological development, internalizing problems, externalizing problems, functioning at home, functioning at school, functioning during leisure time and environment/circumstances. After having investigated all aspects of a domain, a four point Likert type score was given, reflecting the concern by domain (no, some, much, very much). In case of no information, the examiner can fill in *no information*. In order to provide the investigative magistrate with information on the quality of the data for every domain, whether the scoring was based on mono or multi informant data (e.g. adolescent, parent, school, guardian, police) has to be indicated [2].

In addition, in order to support the more or less qualitative information with an empirically based numerical risk score, a discriminant analysis was performed to determine what combination of risk factors collected in the interview and questionnaires best predicted the presence of a psychiatric disorder or psychosocial problems. In total, ten items turned out to possess the required capacity to discriminate between suspects with and without a psychiatric disorder or psychosocial problems (Table 1). The first five items stemmed from interviews with and questionnaires taken from the juvenile offender comprising a Youth-index (Y-index), while the next five items stemmed from the parents' interviews and questionnaires and led to the Parent-index (P-index). Together, ten items formed the Youth & Parent index (Y&P-index). When using the Y-index only, a correct prediction could be obtained for 72% of cases. When using the Y&P-index, 88% of cases were correctly classified [2].

**Table 1:** Index questions

Youth	
1	Do you have trouble in school? (no, yes)
2	Is there extrafamilial violence? (none, mild, moderate, serious)
3	Have you ever been placed out of your home? (no, yes)
4	Do you have pain symptoms? (none, mild, moderate, serious)
5	Do you have problems resulting from alcohol/drugs? (none, mild, moderate, serious)
**Parent(s)**	
6	How many times did the adolescent have contact with the police in the past? (never, 1 time, 2 or 3 times, more than 3 times)
7	Does the adolescent have problems getting along with his/her teachers? (none, some, many)
8	Has the father ever had professional assistance from a mental health agency? (no, yes)
9	Does the adolescent have a history of dangerous behaviour? (never, sometimes, often)
10	How would you describe the mood of the adolescent? (good, somewhat problematic, seriously problematic)

Finally, in collaboration with the CPB, a standard instrument layout was worked out:

1. the *front page *for all relevant personal and (historical) offense related information (standardized and fit for computer processing);

2. the *protocol *describing the rights of the adolescent and caregivers, and regarding the duties of the examiner;

3. the *semi-structured interview *on the nine domains of development and functioning. For reasons of reliability, the aim was to collect information from the adolescent, the caregivers and, if possible, a third informant (e.g. a teacher);

4. the *score sheet *by domain (providing a summary score for all informants);

5. the *Y&P-index *questions with a Likert type score system;

6. the *judicial decision tree *assisting the advisory process;

7. the *report format *layout.

A *user guide *was added [2].

The secondary analysis led to the Dutch version. The instrument was also translated into English, German, Russian and Finnish. It is being used in Switzerland, Austria and Finland. The German-language version was validated in Switzerland [17].

### Current study

After the development of the instrument this study was carried out in order to assess the psychometric properties for psychiatric disorders and psychosocial problems and the perceived usefulness of the BARO.

## Method

### Subjects

In three judicial districts (urban, suburban and rural areas) 295 BAROs were administered. All juveniles were referred to the CPB suspected of committing an offense. Because the BARO was the standard screening instrument for the global assessment, all referred juveniles were consecutively included. For reasons of readability the juveniles will be referred to as offenders, although they were officially only suspected of a criminal offense. Mean age of the subjects was 15.9 years (SD = 1.8; range: 8.5 - 18.9); 91% were boys and 9% girls. The ethnic backgrounds of the subjects were: 57% Dutch, 7% Turkish, 13% Moroccan, 7% Surinamese and 16% 'other'. Most adolescents (95%) lived with (at least one of) their biological parents at the time of their arrest. The majority attended school (94%). Only a small group had had a child protection measure in the past (5%), or had ever been admitted to an inpatient service (6%). Sixty-one percent of the subjects had been accused of a single offense, 31% of two offenses and 8% of three or more offenses. The nature of the offenses was: 48% property offenses, 21% offenses against public order and authorities, 19% assault, 14% aggression against property (e.g. fire setting), 7% sex offenses, and 3% other types of offenses. Forty-one percent of the subjects had a history of registration by the police. Table 2 demonstrates the concern of the CPB worker by domains of the BARO. On most domains there was generally no to some concern. Only a small subgroup is characterized by (very) much concern.

**Table 2:** Concern by domains of the BARO

	No	Some	Much	Very much	No information
	% (N)	% (N)	% (N)	% (N)	% (N)
Delinquent behaviour	18.6 (55)	51.2 (151)	20.0 (59)	5.1 (15)	5.1 (15)
Physical condition	83.1 (245)	12.5 (37)	1.0 (3)	0.3 (1)	3.1 (9)
Psychological development	51.9 (153)	30.8 (91)	12.5 (37)	1.4 (4)	3.4 (10)
Internalizing problems	52.2 (154)	29.8 (88)	10.5 (31)	3.4 (10)	4.1 (12)
Externalizing problems	47.1 (139)	35.9 (106)	11.5 (34)	2.0 (6)	3.4 (10)
Functioning at home	53.2 (157)	21.7 (64)	14.6 (43)	5.8 (17)	4.7 (14)
Functioning at school	38.0 (112)	34.6 (102)	18.3 (54)	2.7 (8)	6.4 (19)
Functioning leisure time	45.8 (135)	35.9 (106)	12.5 (37)	2.7 (8)	3.1 (9)
Environment/circumstances	41.0 (121)	32.2 (95)	19.0 (56)	3.4 (10)	4.4 (13)

Sixty-six offenders (89% boys, 11% girls; 17 court ordered and 49 voluntarily) underwent an elaborate diagnostic assessment by forensic experts (psychologist and child psychiatrist). These sixty-six offenders did not differ from the total BARO sample on age, gender or type of offense. Both the psychologist and the child psychiatrist assigned DSM-IV diagnoses. Final psychiatric diagnostic classification was done during a multidisciplinary consensus meeting. For validation of the BARO completed by the CPB workers, the multidisciplinary forensic expert group (psychologist and psychiatrist) completed the BARO domain score sheet blindly after the diagnostic investigation.

### Procedure

First, the internal consistency of the domain score sheet and the Y-index, P-index and Y&P-index was calculated by means of Cronbach's alpha. Second, the concurrent validity of the instrument was calculated for the participants that underwent the global screening by means of the BARO as well as the elaborate forensic diagnostic assessment. The concurrent validity was based on the correlation of the BARO domain scores (providing a summary score for all informants) assigned by the CPB workers and the forensic experts. In statistical research r < .30 is considered a small correlation, .30 < r < .50 a moderate correlation and r > .50 a strong correlation [18]. However, whether a correlation is poor, sufficient or good depends of the field of research. The discriminatory value of the domain sum score, Y-index and Y&P-index was examined by means of Receiver Operator Characteristics (ROC) estimation. The relationship between the true and false positive rates is demonstrated in a ROC curve, which is a plot of these two rates for every, or given, cut-off point. The area under the curve (AUC) represents a cumulative index of the sensitivity and the specificity at each possible cut-off point. A value of .50 of the AUC indicates chance level and 1.0 indicates a perfect diagnostic tool [19,20]. An AUC < .60 is considered small, .60 < AUC < .80 moderate and AUC > .80 strong [21].

Finally, the perceived usefulness of the instrument was analyzed by descriptive statistics of an additional evaluation form the CPB workers filled out after completing the BARO. This form contained open questions (e.g. What items do you want to be added to the instrument?) as well as multiple-choice questions with Likert type scales (e.g. How useful is the instrument for final advice?). For some responses, four point scales were designed and administered to obtain more quantitative information about the various quality items.

The study was approved by the Dutch Ministry of Justice.

## Results

### Internal consistency

The Cronbach's alpha of the items included in the domain score sheet turned out to be good (0.88). For the Y-index (0.45) and the P-index (0.56) separately, poor Cronbach's alphas were computed. However, the combined Y&P-index Cronbach's alpha was sufficient (0.70).

### Concurrent validity

The concurrent validity of the domain score ratings of the CPB workers and the forensic experts was strong (r = .69; p < .001).

### Discriminatory value

**Figure 1:**
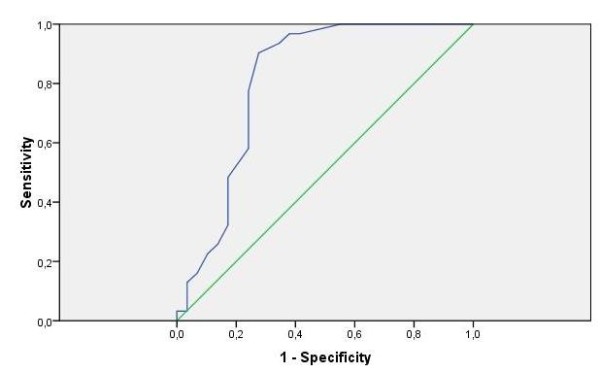
**ROC curve domain sum score on psychiatric disorder**.

The discriminatory value of the domain score, measured by the AUC, was strong, namely 0.81 (95% CI: 0.69 - 0.93, p < 0.0001). Among the different possible cut-off points, the one with optimal sensitivity and specificity was selected. With a cut-off of 6.5, the optimal sensitivity was 77% and the optimal specificity 76% (Figure 1).

**Figure 2:**
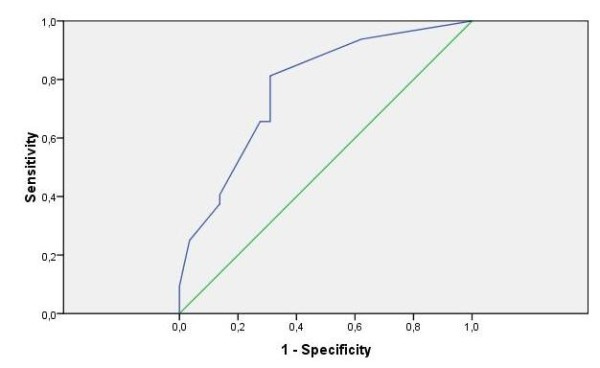
**ROC curve Y-index on psychiatric disorder**.

When using the Y-index only, the AUC was moderate (0.77, 95% CI: 0.66 - 0.89, p < 0.0001). The cut-off of 6.5 was considered optimal, corresponding to a sensitivity of 81% and a specificity of 69% (Figure 2).

**Figure 3:**
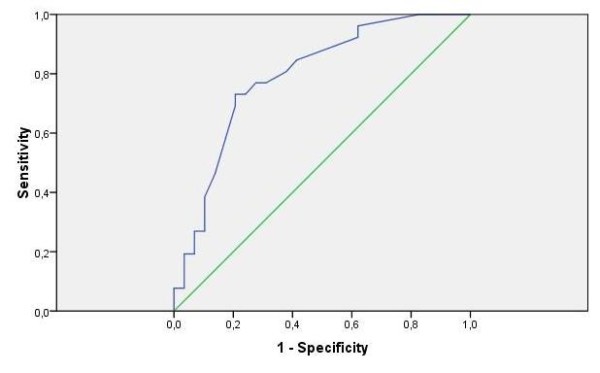
**ROC curve Y&P-index on psychiatric disorder**.

For the Y&P-index, the AUC reached a moderate level of 0.79 (95% CI: 0.67 - 0.91, p < 0.0001). The optimal cut-off of 13.5 had a sensitivity of 77% and a specificity of 72% (Figure 3).

### Perceived usefulness

The majority of the CPB workers evaluated the usefulness of the instrument as good to very good (79.4%), while another 12.2% described the usefulness to be moderate. Only 0.3% of the CPB workers responded that the instrument as a whole was impractical. Most raters examined all domains of disorders and (dys)function (84%), and in the majority of cases, two or more informants had been consulted (89%). In order to formulate the optimal advice, 16.3% of the social workers expressed a need for more information. Seventeen percent of the respondents suggested additional topics (e.g. pedagogical capacities of parents and family history). Concerning the weightings of the domain scores, three quarters of the CPB workers did not mention difficulties.

Almost all CPB workers (N = 285) registered the duration of the BARO administration with the youth, which was on average 74.9 minutes (95% CI: 71.5 - 78.4). The BARO interview with the parents was registered for 248 contacts and took on average 70.4 minutes (95% CI: 67.1 - 73.7). For 87 cases only, was the school contacted, which took on average 22.9 minutes (95% CI: 19.6 - 26.2).

## Discussion

Until the development of the BARO, a standardized screening instrument was not available for CPB workers, resulting in a vast qualitative and quantitative variety of reports. As a result, it was not clear why some juvenile offenders were referred for specialized forensic diagnostic assessment, while others were not. Because of the need to use standardized screening methods and to increase the reliability and validity of the diagnostic work of the CPB, the development of a screening instrument was initiated. As a consequence of the advisory role of the CPB in the juvenile criminal justice system, both objectivity and completeness were necessary, which was achieved by developing a comprehensive interview and a scoring system. The information obtained from the interview results in a written report, according to the preference of the juvenile court, while the scoring system serves as an empirically based risk estimate. One of the tasks of social workers of the CPB is the examination of young offenders in order to detect psychiatric disorders and psychosocial problems and to evaluate the need for treatment. For adequate decision taking with respect to sanctions and treatment, the CPB workers have to provide the judges with a report describing the 'functioning' of the juvenile offender. When psychiatric or psychosocial problems are conjectured, a specialized forensic diagnostic examination can be ordered.

This article describes the reliability, validity and usefulness of the BARO, a first-line screening instrument for juvenile suspects. It was shown that this instrument combines sufficient to good psychometric properties and moderate to strong discriminatory value for psychiatric disorders and psychosocial problems with satisfactory perceived usefulness. First, with respect to reliability, it was demonstrated that information from both the youth and the parent is preferable to results from the youth only. Second, two main screening outcome scores (domain sum score and Y&P-index) showed moderate to strong discriminatory value. And finally, the CPB workers evaluated the BARO as a useful and practicable instrument. The BARO allows the formulation of well-founded advice.

Internal consistency analysis has shown that the Y-index and the P-index perform poorly as separate constructs, whereas this is not so for the Y&P-index. As only 5 items each are included in both the Y-index and the P-index, this is not surprising. Further research should clarify whether the higher consistency is only a consequence of the larger number of items in the combined index, or whether it is related to the advantage of a multi informant approach [22]. The discriminatory value of the Y-index, however, as measured by the AUC, was only slightly lower than for the Y&P-index. This indicates that when parents are not available for screening purposes moderate discriminatory value for detecting psychiatric disorders and psychosocial problems can be obtained from the youth, but not the other way around. As the domain sum score reflects a weighing of information derived from all different informants, psychometric and discriminatory value differences by informants could not be investigated. Future research should focus on this area, because it may help to reduce the amount of information requested from each person, and subsequently the duration and personnel costs of the investigation. Both the domain score sheet and the Y&P-index questions have shown strong discriminatory value. However, it may not be concluded that the Y&P-index is useful as a solitary index, as these index questions were embedded in the complete interview. Hence, it is not clear to what extent the scoring of the Y&P-index items has been influenced accordingly.

Although the psychometric properties and the perceived usefulness of the instrument was considered sufficient to good, a few considerations must be taken into account. First, as it is not known how long a traditional CPB assessment took in the past, it was not possible to compare the duration of BARO administration. In the past, it was neither practice to interview youth and parents separately, nor to interview third party informants. The additional travelling time in particular might have increased the total duration of the assessment. Further adaptations that help to increase the perceived usefulness of the instrument should be considered: (1) qualitative research should investigate whether the BARO content can be reduced (e.g. through telephone interviewing of third party informants), (2) making an electronic BARO version may be helpful, which has been done for the Finnish BARO. Second, in the last decade there has been a major - politically induced - shift in the Dutch juvenile criminal law system from protection of the development of the juvenile offender to protection of society. This also includes a change of focus of screening instruments used by agencies within the criminal justice system. Recent BARO investigations in the Netherlands have shown that the BARO is only of moderate predictive value for reoffending [23]. More research is recommended. Third, rates of offenders living with (at least one of) their biological parents and school attendance were high, whereas rates of a child protection measure or admission to an inpatient service were low. Because it was not known whether these offenders were convicted of their crime, this research was not able to compare suspects to convicted offenders. Future research should focus on this question. Future research should also look into subgroups of juvenile offenders (e.g. gender, ethnic background, mental retardation, specific psychiatric disorders) in more detail. Due to the small sample this was not possible. Fourth, the interrater reliability between CPB workers could not be investigated. Because of the judicial procedure the subjects were involved in, ethical issues did not allow videotaping interviews by CPB workers.

This study has provided evidence that the BARO is a very promising screening instrument for identifying arrested youth at risk for psychiatric disorders and psychosocial problems among adolescents referred to the CPB. As psychiatric disorders occur frequently among delinquent youth, and as it has been demonstrated that these problems frequently go unnoticed, the clinical impact of this finding may not be underestimated [6,11]. Standardized screening may not only bear great impact on the psychosocial well-being of the children and their families through referral to adequate intervention and treatment, but it may also be cost-effective and prevent further antisocial behaviour. These latter aspects, however, need to be investigated in future.

## Competing interests

The authors declare that they have no competing interests.

## Authors' contributions

TD was responsible for the research project. EvL carried out the statistical analyses. RV was co writer of the paper and performed the ROC analyses. CB was co writer and performed all editorial revisions. All authors read and approved the final manuscript.
